# Editorial: Artificial intelligence for child health and wellbeing

**DOI:** 10.3389/fdgth.2025.1685788

**Published:** 2025-09-05

**Authors:** Florian B. Pokorny, Katrin D. Bartl-Pokorny

**Affiliations:** ^1^ Division of Neonatology, Medical University of Graz, Graz, Austria; ^2^ Division of Phoniatrics, Medical University of Graz, Graz, Austria; ^3^ CHI – Chair of Health Informatics, TUM University Hospital, Munich, Germany; ^4^ Munich Center for Machine Learning (MCML), Munich, Germany

**Keywords:** artificial intelligence, machine learning, deep learning, child health, paediatrics, disease detection, intervention, emotion recognition

**Editorial on the Research Topic**
Artificial intelligence for child health and wellbeing

Ongoing rapid advances in digital data collection technology, machine learning methodology, and computer hardware are making artificial intelligence (AI) applications a decisive factor in an ever-expanding range of areas of life. This includes health and wellbeing (1, 2), where AI offers numerous possibilities to optimise daily or clinical routines and, thereby, to improve individuals’ quality of life. Related AI use cases include recognising wellbeing, detecting diseases (at an earlier stage), automatically monitoring disease progression, enabling personalised disease prevention—potentially less bound to hospital stays—or wellbeing preservation, planning and optimising interventions, and evaluating intervention success. While AI has proven promising for tasks related to cancer (3), chronic diseases (4), nervous system diseases (5), and mental diseases (6) in adults, AI is increasingly being applied in context of healthcare and wellbeing of children. This is particularly important, as any improvement at a young age can significantly enhance the long-term outcomes of affected individuals. Specific current applications range from the automatic detection and/or tracking of a variety of medical conditions, such as developmental disorders (7), mood disorders (8), genetic disorders (9), through automatic child emotion recognition (10), to intelligent robot-based intervention for children with autism spectrum disorder (11). Nevertheless, AI for child health and wellbeing remains a niche research area. Therefore, the aim of this Research Topic was to provide insights into the current state of research in this still underrepresented field, to boost further related original work, and to generally highlight the potential of AI in the context of children’s health and wellbeing, while at the same time addressing related ethical aspects and methodology-inherent issues, such as explainability, fairness, potential misclassification, and responsibility.

In the end, four articles were included in this Research Topic. These contributions involved a total of fourteen authors across three continents, representing Austria, Germany, Switzerland, Nigeria, and the United States of America.

The first of the four articles likewise examined the roles that children and adolescents play in the development and implementation of contemporary digital technologies—including AI—for health and wellbeing within one of these three continents, namely Africa. For their policy and practice reviews contribution, Holly et al. first selected ten African countries, in which people under the age of 25 years make up the substantial proportion of the population. Then they identified national digital health strategies for these focus countries and gathered information on different domains, including the consideration of children and youth within the respective strategy. Their findings indicate that national digital health strategies often neglect the specific health needs and distinct risks that digital technologies and data pose to young people. They conclude that future strategies should be designed through inclusive processes that respect and uphold children’s and adolescents’ right to participate in decisions that impact their lives.

In a more technology- and application-centered review article, Bartl-Pokorny et al. provide an overview of automated disease detection studies with a focus on infancy, that is, the very first year of life, published in the five years preceding the release of ChatGPT. They found that the most recent studies, i.e., 46 studies from 2022 focused on medical conditions of twelve different ICD-11 categories with “certain conditions originating in the perinatal period” being the most frequently addressed category. Related examples of diseases are bronchopulmonary dysplasia, necrotising enterocolitis, and hypoxic-ischemic encephalopathy. Bartl-Pokorny et al. further revealed that in most studies, AI models were trained on clinical and demographic information and laboratory data with deep neural networks—not surprisingly—representing the most popular AI approach. Nevertheless, traditional AI methods—such as support vector machines and random forests—still play a significant role, likely due to their advantages in explainability and their effectiveness when available data is sparse. The reported performance of certain AI systems suggests strong potential for automated approaches to support future diagnostic procedures in infants.

A completely different application was addressed in the first of two original research articles of this Research Topic. Khante et al. dealt with the acoustic classification of household chaos—an environmental condition characterised by high levels of noise and crowding alongside a lack of regularity and structured daily routines. The connection to child health stems from the fact that household chaos represents an established risk factor for adverse development; children from more chaotic households are more likely to exhibit behavioral or cognitive deficits. Instruments for measuring chaos, and consequently, children’s risk for suboptimal outcomes, are typically subjective, such as questionnaires completed by caregivers within the households. Khante et al. overcome this issue by introducing an objective, high-resolution (auditory) household chaos level estimation in form of a classifier built upon more than 400 h of selectively annotated, daylong, real-world audio recordings collected via infant-worn microphones. Both their AI model and parts of their dataset were made publicly available to promote further research.

In the second original research article of this Research Topic, Al Futaisi et al. focused on a currently late diagnosed developmental disorder, namely autism spectrum disorder (ASD), and aimed to automatically recognise ASD based on child speech data. A common challenge in speech analysis is the limited availability of large datasets that are annotated for vocalisations. To address this issue, Al Futaisi et al. leveraged two smaller datasets and applied transfer learning techniques through fine-tuning models. They compared two fine-tuning approaches for both a binary classification task (typical development vs. ASD) and a four-class classification task (typical development vs. ASD vs. dysphasia vs. pervasive developmental disorder not otherwise specified). They achieved promising results, suggesting that transfer learning could be a valuable approach for recognising ASD from speech.

The distinctly different topics addressed in the four contributions of this Research Topic demonstrate the breadth of the field of AI for child health and wellbeing, as well as the wide range of related potential applications and implementation-related aspects. Let us be curious to see in which use cases and under which regulatory framework AI systems will make the transition from basic research to clinical or practical application in the coming years in order to improve health and wellbeing of those in our society that are among the most vulnerable—children.
